# Enzymatic Depilation of Animal Hide: Identification of Elastase (LasB) from *Pseudomonas aeruginosa* MCM B-327 as a Depilating Protease

**DOI:** 10.1371/journal.pone.0016742

**Published:** 2011-02-11

**Authors:** Emmanuel Vijay Paul Pandeeti, Gopi Krishna Pitchika, Jyotsna Jotshi, Smita S. Nilegaonkar, Pradnya P. Kanekar, Dayananda Siddavattam

**Affiliations:** 1 Department of Animal Sciences, School of Life Sciences, University of Hyderabad, Hyderabad, Andhra Pradesh, India; 2 Microbial Sciences Division, MACS-Agharkar Research Institute, Pune, Maharashtra, India; Universita di Sassari, Italy

## Abstract

Conventional leather processing involving depilation of animal hide by lime and sulphide treatment generates considerable amounts of chemical waste causing severe environmental pollution. Enzymatic depilation is an environmentally friendly process and has been considered to be a viable alternative to the chemical depilation process. We isolated an extracellular protease from *Pseudomonas aeruginosa* strain MCM B-327 with high depilation activity using buffalo hide as a substrate. This 33 kDa protease generated a peptide mass fingerprint and *de novo* sequence that matched perfectly with LasB (elastase), of *Pseudomonas aeruginosa*. In support of this data a *lasB* mutant of MCM B-327 strain lacked depilatory activity and failed to produce LasB. LasB heterologously over-produced and purified from *Escherichia coli* also exhibited high depilating activity. Moreover, reintroduction of the *lasB* gene to the *P. aeruginosa lasB* mutant *via* a knock-in strategy also successfully restored depilation activity thus confirming the role of LasB as the depilating enzyme.

## Introduction

Leather-making, is a by-product of the meat industry and reduces potential waste as well as contributing to economic growth [1]. Current leather-processing procedures generate a considerable amount of chemical waste during all stages of processing and cause serious environmental pollution [2]. In the conventional pre-tanning process, depilation of animal hide is done by employing lime and sulphide. These two chemicals alone account for 70% of the total pollution in terms of biological oxygen demand (BOD), chemical oxygen demand (COD), total dissolved solids (TDS) and total suspended solids (TSS) [3]. The alkaline nature of tannery effluents and the high sulphide content pollute ground water sources and cause serious health problems to the tannery workers and people living in the vicinity of leather-processing industries [4], [5].

A number of attempts have been made to find alternative methods for depilation of animal hide. The use of microbial enzymes, especially extracellular proteases have proved to be highly effective in depilating of animal hides [6]. Though a number of bacterial and fungal strains are known to grow on hides, only a few of them have been shown to produce extracellular proteases with depilatory activity [7], [8], [9], [10], [11]. In principle, the proteases having high depilatory properties with mild or no collagenolytic activity are considered to be the best proteases for depilating animal hide [12], [13], [14], [15], [16].

*Pseudomonas aeruginosa* MCM B-327 isolated from vermicompost pit soil has been shown to produce a depilating protease with no significant collagenolytic activity and its potential use in depilating buffalo hide has been successfully demonstrated [17]. The use of strain MCMB-327 is restricted to large-scale production of the depilating protease since *P. aeruginosa* is an opportunistic pathogen. If the candidate gene coding for the depilating protease could be identified and expressed in any one of the GRAS (Generally Regarded As Safe) organisms, the recombinant enzyme could be safely used to substitute for chemical treatment in the leather industry. In this study, we report the cloning of a candidate gene coding for the depilatory protease and present evidence using gene knock-out and knock-in strategies, showing that the depilatory activity is due to the product of the *lasB* gene. Heterologously expressed and purified LasB and a variant secreted as an extracellular protein successfully depilated buffalo hide, showing its utility in the leather industry.

## Materials and Methods

### Media and bacterial growth conditions

The bacterial strains and plasmids used in this study are given in Table 1. Unless otherwise stated *Pseudomonas aeruginosa* MCM B-327 was grown on nutrient agar medium at 30°C. However, for optimal depilating enzyme activity *P. aeruginosa* MCM B-327 was grown on tryptone-soy medium (pH 7.0), which typically contains 1% (w/v) tryptone and 1% (w/v) soyabean meal. *E. coli* was grown in LB medium (pH 7.0) at 37°C. When required, the antibiotics ampicillin (100 µg/ml), kanamycin (50 µg/ml), gentamycin (10 µg/ml) and chloramphenicol (30 µg/ml) were added to the growth medium at the indicated concentration.

**Table 1:** Bacterial strains and plasmids.

Strain/Plasmid	Genotype or phenotype	Reference
*P. aeruginosa* MCM B-327	Am^r^, Cm^r^ wild type strain	[17]
*P. aeruginosa* MCM B-327-B1	Amp^r^, Cm^r^Gm^r^ Derivative of wild type *P. aeruginosa* MCM B-327 strain generated by replacing *lasB* with *lasB::gm*	This work
*P. aeruginosa* MCM B-327-B2	Amp^r^, Cm^r^,Gm^r^ , Km^r^, LasB negative mutant of *P. aeruginosa* MCM B-327-B1 with pMMBD	This work
*E.coli* DH5α	*supE*44 *ΔlacU*169 (ϕ80 *lacZ ΔM*15) *hsdR*17 *recA*1 *endA*1 *gyrA*96 *thi*1 *relA*1	[18]
pGEMT-Easy	Amp^r^, Cloning vector with 5′ T overhangs	Promega
pMMB206	Cm^r^, Broad host range mobilzable vector	[19]
pMMB-Km	Km^r^, broad host range expression vector created by replacing cm^r^ gene with Km^r^ gene in pMMB206.	This work
pUC18	Amp^r^, high copy number cloning vector	[20]
pSUP202	Amp^r^, Cm^r^, Tc^r^, Mob^+^	41
pGEMD	Amp^r^, 2 kb *lasB* fragment cloned in pGEMT-Easy vector.	This work
pGEMD153	Amp^r^, 2 kb *lasB* mutant coding for LasB T-153I in pGEMT-Easy vector	This work
pMMBD	Km^r^, 2 kb *lasB* cloned in pMMB-Km vector.	This work
pUCD	Amp^r^, 2 kb *lasB* cloned in pUC18 vector as an *EcoR*I fragment.	This work
pUCDG	Amp^r^, Gm^r^ , *lasB*::*gm* cloned in pUC18	This work
pSUPDG	Amp^r^, Gm^r^,Tet^r^, *lasB*::*gm* cloned in pSUP202 as an *EcoR*I fragment.	This work

### DNA manipulations

All plasmids and genomic DNA used in this study were isolated using Qiagen miniprep / DNAeasy kits following the manufacturer's protocols. Restriction enzymes and T4 DNA ligase were purchased from MBI Fermentas. Routine DNA manipulations were performed following standard procedures described elsewhere [22].

### Production of protease

*Pseudomonas aeruginosa* MCM B-327 was grown in tryptone-soyabean medium (pH 7.0) for 72 h at 30°C with rotary shaking at 200 rpm. After incubation, the culture was centrifuged at 15000g for 20 min to remove cells. The spent medium was saturated with 60% ammonium sulphate. Ammonium sulphate precipitate was dissolved in 50 mM Tris buffer (pH 8.0) and dialysed against five liters of the same buffer.

### Protease assay

The protease activity was determined by following standard protocols with slight modifications [23]. To measure protease activity in the spent medium, the medium was mixed in 1∶ 4 ratio with freshly prepared casein (0.625% w/v) solution and incubated at 37°C for 30 min. The reaction was stopped by addition of 5 ml of 5% (w/v) trichloroacetic acid and the protease activity was measured by determining the rate of enzymatic hydrolysis of casein by the Folin Ciocalteu method [24], [25]. One unit of protease activity was defined as the amount of enzyme required to liberate 1 µg of tyrosine per min at 37°C.

### Casein agar plate assay

Secretion of extracellular protease was monitored by performing a casein-agar plate assay. Colonies to be tested for their ability to secrete extracellular protease were streaked on casein containing agar plates prepared by using autoclaved casein agar solution (1.5% w/v), or skimmed milk powder and agar (2%) in Tris-HCl buffer (50 mM, pH 7.0). After streaking, the plates were incubated at 30°C and were frequently monitored for the formation of a clear zone due to hydrolysis of the casein. If a clear zone was formed within 6 h of incubation it was taken as an indication of the secretion of extracellular protease.

### Assay of depilating activity

The depilating activity of the extracellular protease was tested on a fresh animal hide obtained from a local slaughterhouse. Initially the thoroughly washed fresh animal hide was cut into 2 cm^2^ pieces and soaked with 50 mM Tris-Cl (pH 8.0) after placing them separately in a sterile petri-dish. The plates were then incubated for 16 h after applying 200 U of enzyme uniformly to the animal hide. After incubation, the hair from the hide was scraped with a sterile spatula to monitor depilating activity. The hide piece treated with 50 mM Tris buffer (pH 8.0) instead of protease served as negative control.

### Development of a zymogram assay on native PAGE

About 200 µg of total ammonium sulfate-precipitated protein was resuspended in sample buffer (50 mM Tris, pH 8.0, 5% w/v sucrose, 0.1% w/v bromophenol blue) before loading a portion on two identical native 7.5% polyacrylamide gels [26]. After electrophoresis, one of the gels was stained with Coomassie Brilliant Blue R-250 and the other was used for developing a zymogram. The unstained gel was soaked in 1% (w/v) casein solution for 1 h and thoroughly washed with distilled water before staining with Coomassie Brilliant Blue R-250. The gel was then destained and clear zones generated due to proteolytic activity were documented by scanning using an Imagescanner (GE Healthcare).

### Purification of depilating enzyme

The dialyzed ammonium sulphate precipitate was loaded on a manually packed DEAE sepharose column equilibrated with 50 mM Tris-Cl (pH 8.0) containing 0.18 M NaCl. The protein was eluted at a flow-rate of 0.5 ml/min with a linear gradient of 1 M NaCl in 50 mM Tris buffer (pH 8.0). All fractions obtained from DEAE chromatography and the flow-through were tested for depilating activity. The active fractions were analyzed by SDS-PAGE [27].

### Gel Permeation Chromatography

The protein found in the active fractions was concentrated by re-precipitating with ammonium sulphate (60% saturation) and was dissolved in 5ml of 50 mM Tris buffer (pH 8.0) and loaded onto a manually packed Sephacryl 200 HR column (XK 16/100) fitted to an AKTA basic FPLC system (GE Healthcare). The column was equilibrated with 7 column volumes of 50 mM Tris buffer (pH 8.0) and chromatography was performed at a flow-rate of 0.5 ml/min. The fractions collected were tested for depilatory activity and the active fractions were analyzed on SDS-PAGE [27].

### Two Dimensional gel electrophoresis

All chemicals and IPG strips used for two-dimensional (2D) electrophoresis were obtained from GE Healthcare, USA. 100 µg of pure depilating enzyme obtained from gel permeation chromatography was precipitated with methanol-chloroform (4∶1) and dissolved in 350 µl of sample buffer (7 M urea, 4% (w/v) CHAPS, 2% (v/v) pharmalyte, 40 mM DTT) before loading on IPG strips (18 cm, pH 3–10). The strips were then actively rehydrated for 12 h at 20 V and IEF was performed on an Ettan IPGphor3 system using a four-step programme [500 V for 30 min (gradient), 500 V for 30 min (step), 8000 V for 3 h (gradient); and electrophoresis was continued for 40,000 Vh]. After focusing, strips were equilibrated with equilibration buffer I (75 mM Tris, 6 M urea, 2% (w/v) SDS, 20% (v/v) glycerol, 2% (w/v) DTT) and then with equilibration buffer II (75 mM Tris, 6 M urea, 2%(w/v) SDS, 20%(v/v) glycerol, 2% (w/v) idoacetamide) each for 15 min. The second dimension electrophoresis was performed on 12.5% (w/v) denaturing polyacrylamide gels in an Ettan DALTsix system (GE Healthcare) at a constant voltage of 200 V. The gels were stained with Coomassie Brilliant Blue R-250 and results were recorded by scanning the stained gel before picking the spots.

### MALDI-MS

The protein spots were processed and digested with trypsin following procedures described elsewhere [28]. The tryptic peptides were dissolved in 2 µl of 50% (v/v) acetonitrile (ACN) containing 1% (w/v) trifluoroacetic acid (TFA) and mixed with 2 µl of 1% (w/v) cyano-4-hydroxycinnamic acid (HCCA) dissolved in 50% (w/v) ACN and 1% (w/v) TFA. Finally, 1 µl tryptic peptides were mixed with 1% (w/v) HCCA was applied to the MALDI target plate. Peptides were analyzed using MALDI-TOF-TOF Autoflex (Bruker Daltonics) in reflectron mode. MS/MS of selected peptides was performed by LIFT. The spectra were calibrated by Pepmix (Bruker Daltonics).

### Protein Identification

The spectral data were analyzed using Biotools software and searches were performed for protein identification using the MASCOT search engine (www.matrixscience.com) against the Swiss-Prot (http://www.expasy.ch/sprot) and the NCBInr (http://www.ncbi.nlm.nih.gov/) databases. The following search parameters were used: trypsin is the enzyme used and one missed cleavage was allowed, the peptide tolerance was set at ±0.5 Da, carbamidomethylation and oxidized methionine were set as fixed and variable modifications, respectively. MS/MS data was analyzed using Biotools software and a mass tolerance of ±0.2 Da was used.

### Amplification and cloning of the gene encoding the depilating protease

The sequence of the *lasB* gene along with its upstream region was taken from the *Pseudomonas* genome database (www.pseudomonas.com) and primers DHEF (5′-CTAGCTGCCACCTGCTTTTC-3′) and DHER (5′-TGAACTTTAGACCGGGTTCG -3′) were designed using the Primer3 software (http://frodo.wi.mit.edu/). PCR was performed using the genomic DNA of *P. aeruginosa* MCM B-327 as the template. The conditions used were 95°C for 5 min, followed by 30 cycles of 95°C for 1 min, 56°C for 1 min and 72°C for 2 min and an extension at 72°C for 20 min. The 1.5 kb amplicon was cloned into the pGEMT-Easy vector (Promega) and recombinant clones were confirmed by restriction digestion followed by DNA sequencing.

### Generation of *lasB* mutants of *P. aeruginosa* MCM B-327

The complete *lasB* gene was isolated as an *Eco*RI fragment and ligated into pUC18 digested with *Eco*RI to obtain pUCD. In recombinant plasmid pUCD, there is an unique *Not*I site in the coding sequence of *lasB*. In order to generate insertionally inactivated *lasB* (lasb::gen) the gentamycin gene was obtained as *Not*I fragment from plasmid pGEN150 (our unpublished work) and ligated into a *Not*I digested pUCD plasmid. The *lasB::gm* gene was isolated as an *Eco*RI fragment and ligated into the mobilizable suicide vector pSUP202 previously digested with *Eco*RI to obtain pSUPDG. *E.coli* S17-1 harbouring pSUPDG was used as donor to mobilize *lasB::gm* into *P. aeruginosa*. Conjugation was performed using standard procedures [29]. Exconjugants were selected on plates containing Km (50 µg/ml) and Gm (10 µg/ml). The exconjugants were then screened for proteolytic activity by plating them on casein-agar plates. Replacement of *lasB* with *lasB::gm* through homologous recombination was confirmed by performing PCR using *lasB*-specific primers.

### Expression of LasB variant

A mutant of *lasB* encoding a LasB variant in which the threonine at amino acid position 153 was substituted with isoleucine was generated by PCR mutagenesis [30]. Plasmid pGEMD was used as template, the forward primer (5′-CCGGTCATCTTGCAAGCCGCGGTC-3′) contained the desired mutation and the reverse primer (5′-GACCGCGGCTTGCAAGATGACC GG-3′) was complimentary to the forward primer. The PCR reaction was performed for 18 cycles following procedures described elsewhere [30]. The PCR product was then digested with *Dpn*I before transforming plasmids into *E. coli* DH5α. The colonies that appeared on an ampicillin plate were replica-plated onto casein-agar plates to identify colonies that gave a clear zone due to production of extracellular LasB. Plasmids isolated from these colonies were sequenced to confirm the presence of the desired mutation in *lasB* gene.

## Results

In *P. aeruginosa* MCM B-327, secretion of the depilating enzyme initiated only in early stationary phase (after 48 h of incubation) and its accumulation in spent medium showed a steady increase till the culture reached the late stationary phase (72 h of incubation). Further incubation had no influence on production of the depilating activity [17]. Interestingly, along with the depilating enzyme activity a bluish-green pigment accumulated in the medium, which turned brown on further incubation giving a greenish-brown appearance to the spent medium.

### The depilating enzyme is an extracellular protease

**Figure 1:**
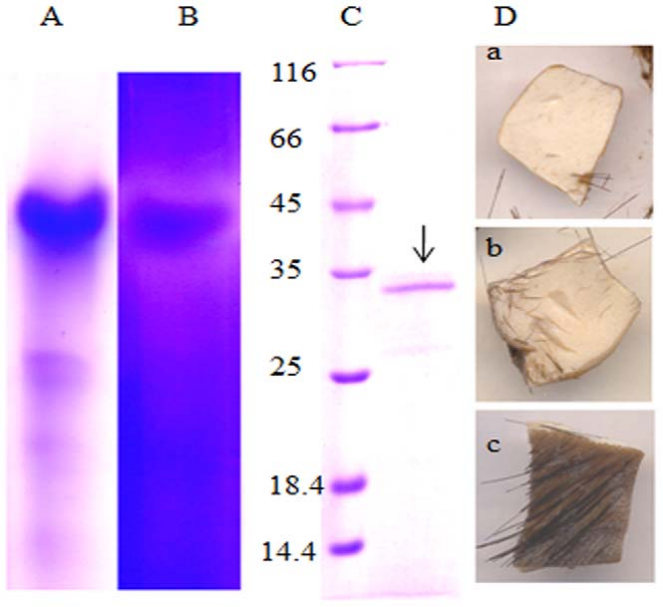
Identification of depilating protease of *Pseudomonas aeruginosa* MCM B-327. Panel A and B represent native PAGE and the corresponding zymogram of extracellular proteins of *Pseudomonas aeruginosa* MCM B-327. Panel C. SDS-PAGE showing the molecular mass of the depilating protease electro-eluted from the zymogram. Lane 1 represents protein molecular mass markers. The 33 kDa depilating protease band found in lane 2 is shown with an arrow. Depilating activity shown by crude extracellular protease (a), protease electro-eluted from the zymogram (b) and a control hide kept by adding buffer instead of protease are shown in panel D.

The spent medium collected from a culture grown for 72 h was initially brought to various levels of saturation with ammonium sulphate. The proteins precipitated during each stage of saturation were independently tested for both protease and depilating activity. Only the protein precipitate obtained from the spent medium saturated to 60% ammonium sulphate showed the presence of a protease with depilating activity. A zymogram developed for the proteins found in this fraction revealed the existence of a single clear zone around a major protein band (Figure 1 A & B). No other clear zones were seen in the entire zymogram, indicating the existence of a single protease complex in the 60% ammonium sulphate precipitate. To gain further insights into the depilating activity the protein band tested to be protease-positive in the zymogram was electro-eluted from the native gel and was applied to a fresh buffalo hide. The electro-eluted protein gave a single band of 33 kDa on SDS-PAGE and successfully depilated animal hide providing direct evidence for the depilatory properties of the extracellular protease (Figure 1C & D).

### Purification of the depilating protease

**Figure 2:**
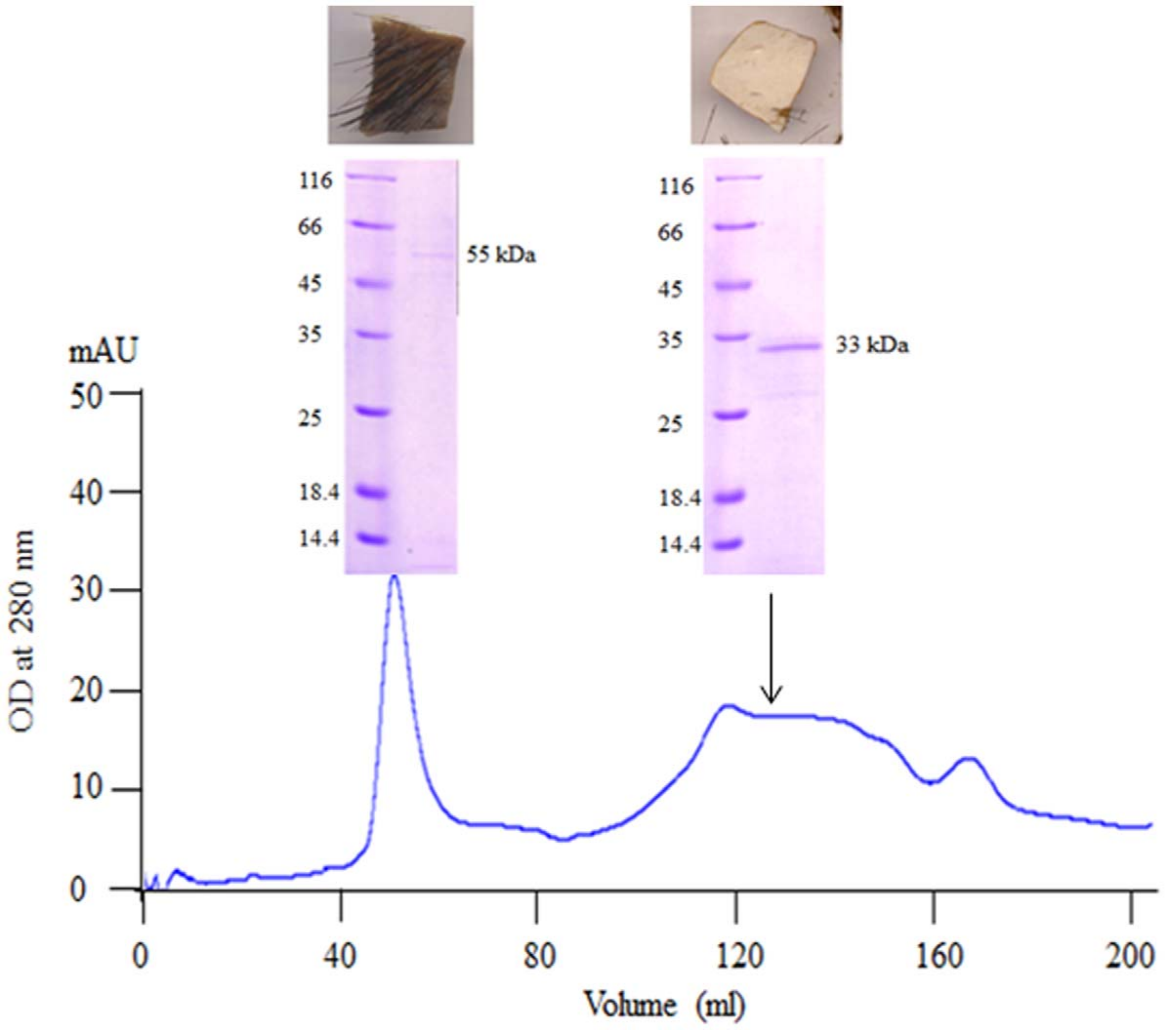
Purification of extracellular depilating protease from *Pseudomonas aeruginosa* MCM B-327: Peak I and II show elution of 55 kDa and 33 kDa proteins from the gel filtration column. Depilating activity is found only with 33 kDa protein.

After identifying the depilating enzyme as an extracellular protease of 33 kDa, further experiments were done to establish its identity. Initially the extracellular protease was purified using conventional ion-exchange chromatography. When loaded onto a DEAE anion-exchange column most of the depilatory protease activity was found in the flow through. However, the impurities that contributed to the brown colour in the culture filtrate and other proteins with no protease activity bound to the DEAE column. Therefore, the depilatory protease activity found in the flow-through, along with other impurities, was concentrated by precipitating with ammonium sulphate (60%). The proteins found in the precipitate were then separated on a Sephacryl S-200 gel-filtration column to achieve further purification. After chromatography, two major peaks were obtained and they were designated as peak-I and peak-II (Figure 2). When proteins found in these peaks were analyzed on SDS-PAGE, a 54 and 33 kDa proteins were found in peak I and peak II, respectively. Further, proteins found in both peak-I and peak-II were independently tested to determine which one of these two peaks possessed depilatory activity. The 54 kDa protein obtained from peak-I showed no depilatory activity, whereas the 33 kDa protein found in peak-II showed a strong depilatory activity. The size of the protein matched the size of the depilating protease electro-eluted from the zymogram (Figure 1C) providing primary evidence to show that the purified protein was the depilating protease that gave a clear zone in the zymogram.

### Identification of the depilating protease

The purified, depilating enzyme was subjected to two-dimensional PAGE to determine the purity of the depilating protease. Three closely associated protein spots were found on 2D gels in the 33 kDa range (Figure S1, panel A). All three closely associated spots were observed in the pI range of 6.5–7.0. In order to identify these polypeptides each spot was manually excised and subjected to trypsin digestion. MALDI-TOF and MALDI-TOF-TOF were performed to obtain peptide mass fingerprint (PMF) (Figure S1, panel B) and MS/MS data (Figure S1, panels C–F). All three spots showed identical PMF profiles suggesting that the three closely associated spots on 2D gels were products of the same gene. The observed differences in pI values of the protein spots might be due to posttranslational modifications. Further, when the peptide mass profiles were used to search the protein database significant scores were obtained for elastase, the product of the *lasB* gene of *P. aeruginosa*. The MS/MS data obtained for four prominent m/z peaks showed significant scores for LasB (Figure S1, panels C–F), further confirms that the depilating protease as the product of the *lasB* gene of *P. aeruginosa* MCM B-327.

### The depilating protease is the product of the *lasB* gene

**Figure 3:**
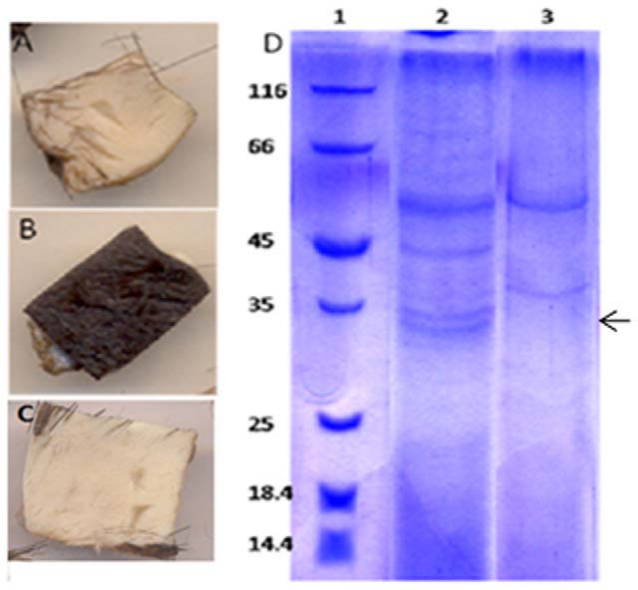
Depilating of animal hide by extracellular proteins obtained from *P.aeruginosa* MCM B-327 (panel A) *P.aeruginosa* MCM B-327-B1 (panel B) and *P.aeruginosa* MCM B-327-B2 (panel C). Extracellular protein profile of *P. aeruginosa* MCM B-327 (lane 2) and knockout strain (lane 3) is shown in panel D. Absence of the 33 kD LasB is shown with an arrow.

After establishing the identity of the polypeptides, a parallel approach was taken to prove the role of the *lasB* gene product in depilation of animal hide. The first approach was to test if the depilating protease is produced by the *lasB* knockout strain of *P. aeruginosa* MCM B-327, while the second one was to demonstrate depilating activity with the heterologously produced LasB. The *lasB* mutant was generated by introducing the insertionally inactivated *lasB* into *P. aeruginosa* MCM B-327 as described in the methods section [21]. In most of the gentamycin-resistant exconjugants, the wild-type *lasB* gene was replaced with the *lasB*::*gm* insertion through homologous recombination. PCR amplification using *lasB*-specific primers gave an amplicon whose size was similar to that of *lasB*::*gm* (data not shown). The *lasB* mutant was then used to check if it produced depilating activity. As expected, in the *lasB* mutant, neither depilating activity nor protease activity could be detected in the spent medium (Figure 3B). Further, no 33 kDa polypeptide corresponding to the extracellular protease was seen on SDS-PAGE confirming that the *lasB* mutant of *P. aeruginosa* failed to produce the depilating protease (Figure 3D).

**Figure 4:**
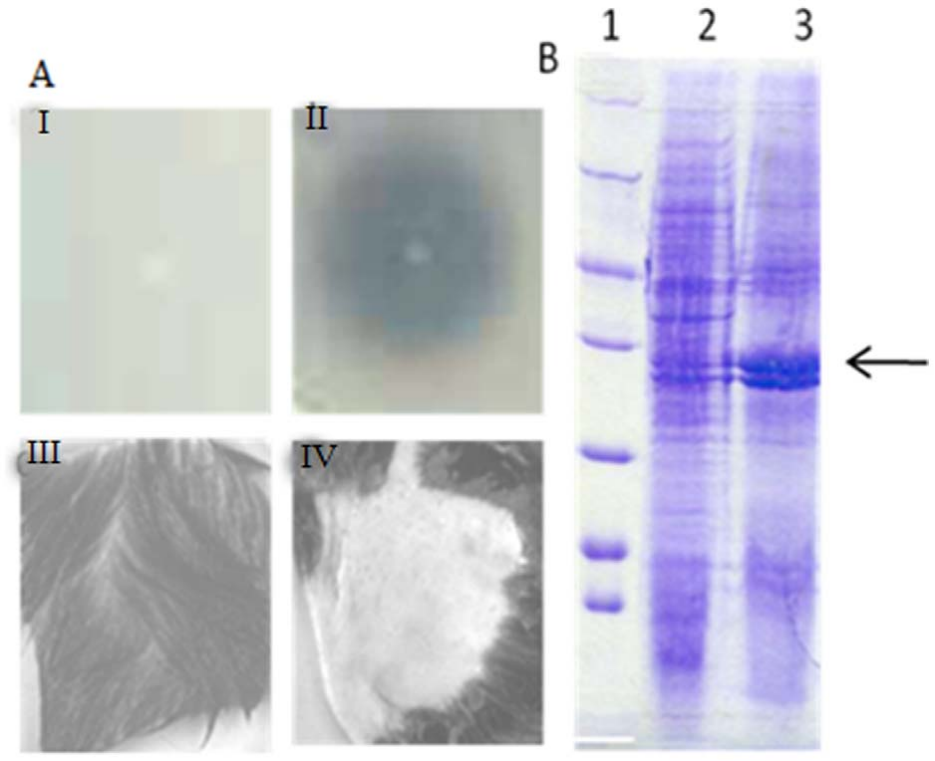
Panel A shows caseinolytic activity of (I) *E.coli* DH5α (pGEMD) and (II) *E.coli* DH5α (pGEMD153) and depilating activity of extracellular proteins produced by *E. coli* DH5α (pGEMD) (III) and *E. coli* DH5α (pGEMD153) (IV) Panel B shows SDS-PAGE profile of extracellular proteins of *E.coli* DH5α (lane 1) and *E.coli* DH5α (pGEMD153) (lane 2).

To test for gain-of-function, *E. coli* DH5α was transformed with plasmid pGEMD in which the *lasB* gene of *P. aeruginosa* MCM B-327 was expressed from its own promoter. Growth of these cultures was very slow and our attempts to achieve maximum growth by prolonged incubation of the culture resulted in cell lysis (data not shown). The cell lysate prepared from these cultures had a significant amount of caseinolytic and depilating activities, suggesting successful expression of active *lasB* in *E. coli* (pGEMD) (data not shown). However, when these cultures were plated on casein-agar no clear zone was observed indicating that LasB was not excreted as an extracellular protein (Figure 4A (I)). Previous studies revealed that introduction of a Thr153Ile substitution resulted in secretion of LasB in *E. coli* as an extracellular protein [31]. Therefore, we generated such a variant by substituting threonine at amino acid position 153 with isoleucine (see methods section). The *E.coli* cells expressing the LasB variant, as determined by extracellular caseinolytic activity, produced a significant amount of extracellular LasB (Figure 4A (II) & 4B). When tested, the LasB variant successfully depilated animal hide at a level comparable with the wild-type LasB produced by *Pseudomonas aeruginosa* MCM B-327 (Figure 4A (IV)).

The loss of depilating activity in the *lasB* mutant of *P. aeruginosa* MCM B-327 and the gain of depilating function by an *E. coli* strain, which otherwise produce no depilating proteases, provide substantial evidence for the involvement of LasB in depilating animal hide. In order to provide final confirmation, a *lasB* knock-in strain, *P. aeruginosa* MCM B-327-B2 was generated by mobilizing the *lasB* gene cloned in a broad-host range vector into the *lasB* mutant B-327-B1 [29]. The knock-in strain of *P. aeruginosa* MCM B-327-B2 restored both production of extracellular LasB and depilating activity providing strong evidence to show that LasB is responsible for the depilation of animal hide in *Pseudomonas aeruginosa* MCM B-327 (Figure 3C).

## Discussion

Protein secretion in prokaryotes, especially in pathogenic *P. aeruginosa* has attracted attention of a number of investigators working to understand host-pathogen interactions [32], [33], [34]. According to an *in silico* prediction, nearly 19.4% of the total proteome is exported into the extracellular milieu by using various protein secretion pathways. Among the extracellular proteins, proteases play a predominant role in colonizing the host after infection [35]. In *P. aeruginosa*, LasB represents nearly 60% of the extracellular protein and generates multiple forms during the course of its maturation [36], [37]. Moreover, *lasB* is one of the virulence factors whose expression is regulated by a quorum-sensing signal molecule N-acyl homoserine lactone [38], [39].

Extracellular proteases of *P. aeruginosa* have been used in a number of industrial applications [40], [41]. They are given different names based on the assay substrates used for monitoring of activity [42], [43], [44]. Recent studies have successfully demonstrated use of extracellular protease in degradation of feather waste generated from the poultry industry [45]. Our earlier studies have clearly shown involvement of an extracellular protease of *P. aeruginosa* MCM B-327 in depilating animal hide without causing any damage to the leather [17]. The present study has identified the extracellular protease as the product of *lasB*. The *lasB* knock-out and knock-in strains of *P. aeruginosa* have shown convincingly that the depilating protease is LasB. In the light of these studies we have revisited the work done by Lin and his coworkers [18]. The authors have successfully shown involvement of an extracellular protease of *P. aeruginosa*, designated as keratinase, in solubilization of feather waste from poultry industry. The sequence of the corresponding gene presented by the authors was aligned with the sequence of LasB reported in this study. Interestingly, 100% similarity was seen between these two proteins suggesting that keratinase reported by them is LasB [42]. The N-terminal sequence of the mature protein (AEAGGPGG), the signal peptide and the pro-peptide coincide perfectly with the LasB sequence reported in the present study.

The quorum-sensing signaling mechanism is known to influence expression of genes that affect cellular physiology in a number of ways. LasB expression has been shown to be under the control of 3OC12-HSL [46]. Binding of the 3OC12-HSL-LasR complex to the sites designated as OP1 and OP2 boxes found upstream of the *lasB* promoter were shown to influence positively its expression during early and late stationary phase [47]. This *lasB* expression pattern coincides with our observation, where accumulation of LasB was shown in the spent medium collected from early and late stationary phases. This evidence further supports our observation that the depilating enzyme is LasB.

The results of heterologous expression studies presented here differ slightly from the published reports, where LasB is shown expressed as an active extracellular protein [43], [48]. In our experience, the extracellular LasB produced in *Pichia pastoris* was found to be inactive. Further, certain recent studies have shown expression of LasB in *E.coli* as an extracellular protein [42], [44]. In our experience, the *lasB* gene cloned in pGEMT-Easy has produced considerable amount of LasB. However, most of it accumulated inside the cells causing growth inhibition and subsequent cell lysis. In our study, only the LasB variant, LasB T153I was secreted effectively into the spent medium (Figure 4B). There is no significant difference in the depilating activity of LasB produced either from the wild-type strain of *P. aeruginosa* MCM B-327 or from *E. coli*. Both LasB and its variant have shown identical depilating properties. As the LasB variant was secreted as an extracellular protein in *E. coli* it can be successfully used for large-scale production and subsequent application in the leather-processing industry.

## Supporting Information

Figure S1 **Analysis of depilating protease of** ***Pseudomonas aeruginosa*** **MCM B-327 by TwoD electrophoresis (Panel A).** Peptide mass fingerprint (PMF) of depilating protease is shown in panel B. Panels C to F represent MS/MS profiles of peaks with m/z values of 1167.604, 1610.825, 2082.871 and 2560.216, respectively.
(TIF)
