# Supplementary figures and images for: Enzymatic Depilation of Animal Hide: Identification of Elastase (LasB) from Pseudomonas aeruginosa MCM B-327 as a Depilating Protease
Source: PLoS One. 2011 Feb 11;6(2):e16742. doi: 10.1371/journal.pone.0016742 (PMC3037957; doi:10.1371/journal.pone.0016742)

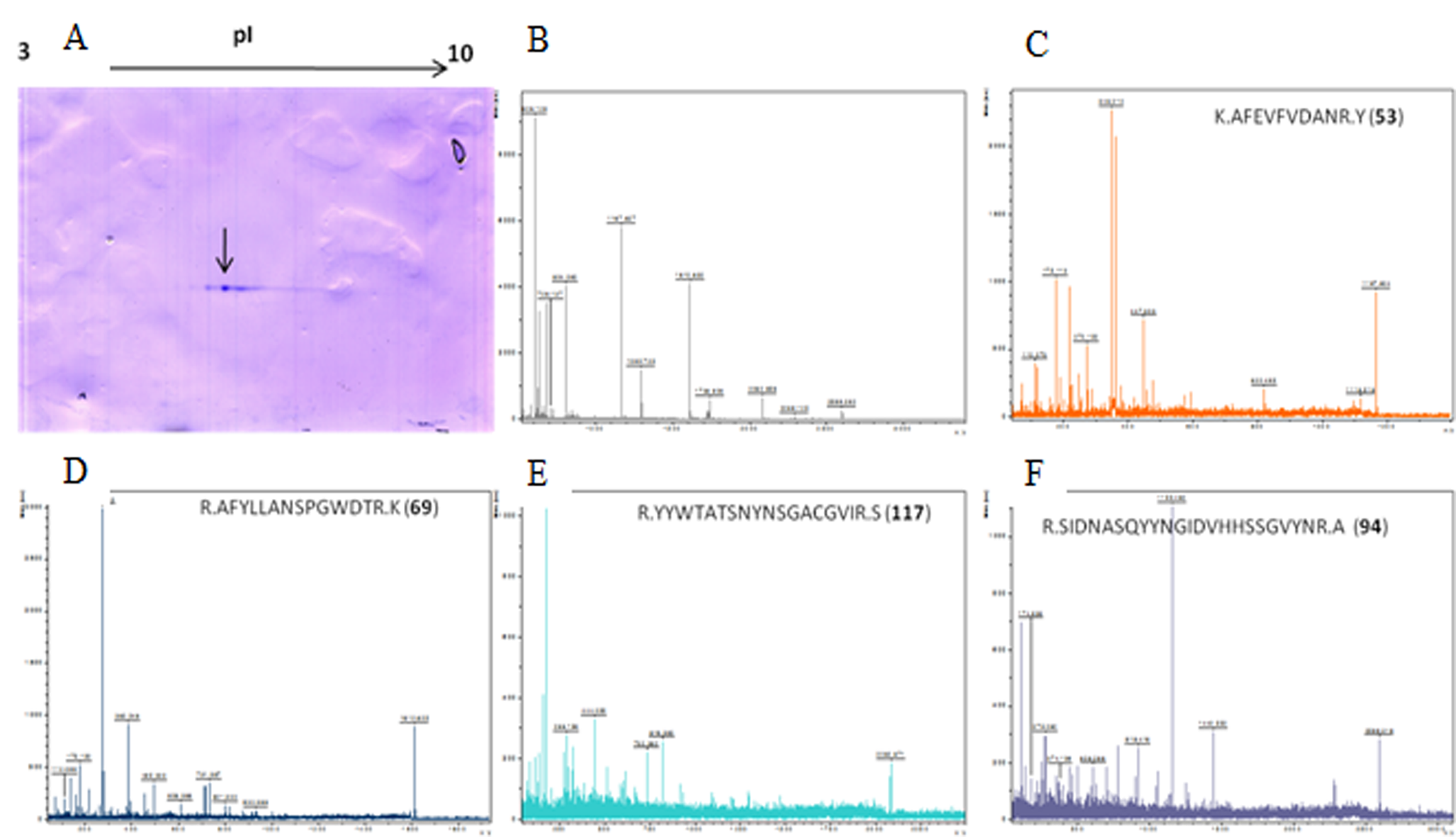

Supplement: Figure S1 — Analysis of depilating protease of Pseudomonas aeruginosa MCM B-327 by TwoD electrophoresis (Panel A). Peptide mass fingerprint (PMF) of depilating protease is shown in panel B. Panels C to F represent MS/MS profiles of peaks with m/z values of 1167.604, 1610.825, 2082.871 and 2560.216, respectively. (TIF) [file pone.0016742.s001.tif]
